# Axes Mapping and Sensor Fusion for Attitude-Unconstrained Pedestrian Dead Reckoning †

**DOI:** 10.3390/s26061968

**Published:** 2026-03-21

**Authors:** Constantina Isaia, Lingming Yu, Wenyu Cai, Michalis P. Michaelides

**Affiliations:** 1 Department of Electrical Engineering, and Computer Science and Engineering, Cyprus University of Technology, Limassol 3036, Cyprus; cn.isaia@edu.cut.ac.cy (C.I.); ly.yu@edu.cut.ac.cy (L.Y.); 2 School of Electronics and Information Engineering, Hangzhou Dianzi University, Hangzhou 310018, China; caiwy@hdu.edu.cn

**Keywords:** pedestrian dead reckoning (PDR), smartphone sensors, step detection, heading estimation, axis mapping, sensor fusion, Wi-Fi fingerprinting, attitude-unconstrained

## Abstract

Localization and navigation techniques have become fundamental for modern lives, while achieving accurate results indoors still remains a significant challenge. The widespread adoption of smart devices, and especially smartphones, has increased the need for accurate and robust pedestrian dead reckoning systems that operate in infrastructure-less environments. Pedestrian dead reckoning’s primary challenge is maintaining accuracy despite varying smartphone placements (attitudes) and the noisy, low-cost inertial measurements units. In this work, a comprehensive pedestrian dead reckoning framework is presented that integrates advanced step counting and heading estimation techniques. For step detection and counting, we propose a robust step counting algorithm that utilizes the optimum fusion of the raw IMU readings, i.e., accelerometer, linear accelerometer, gyroscope, and magnetometer readings, each broken down into three degrees of freedom for different body placements and walking speeds. Furthermore, to address the critical issue of heading estimation, we propose the heading estimation axis mapping (HEAT-MAP) algorithm, which dynamically adjusts the sensor axes in response to the smartphone’s orientation, ensuring a consistent coordinate frame and reducing heading drift. Moreover, to eliminate cumulative pedestrian dead reckoning errors, the system incorporates an adaptive weighted fusion mechanism with Wi-Fi fingerprinting. Experimental results demonstrate that this integrated system significantly improves the overall trajectory accuracy, providing a high-precision, attitude-unconstrained solution for real-time indoor pedestrian navigation.

## 1. Introduction

The evolution of location-based services (LBS) has made seamless positioning and navigation a fundamental requirement of modern society. While global navigation satellite systems (GNSS) provide real-time positioning accuracy to within a meter in outdoor environments, their effectiveness is significantly reduced indoors due to signal attenuation, multipath propagation, and non-line-of-sight (NLoS) conditions [1]. As the number of people enjoying daily activities indoors increases, accurate indoor localization has become essential for applications such as emergency response, smart buildings, and navigation assistance. Indoor positioning is one of the most active research topics in wireless communications and sensor fusion, according to numerous survey studies. For instance, the authors in [2,3,4] provide comprehensive reviews of indoor localization techniques, demonstrating the limitations of GNSS indoors and the need for alternative solutions.

**Figure 1:**
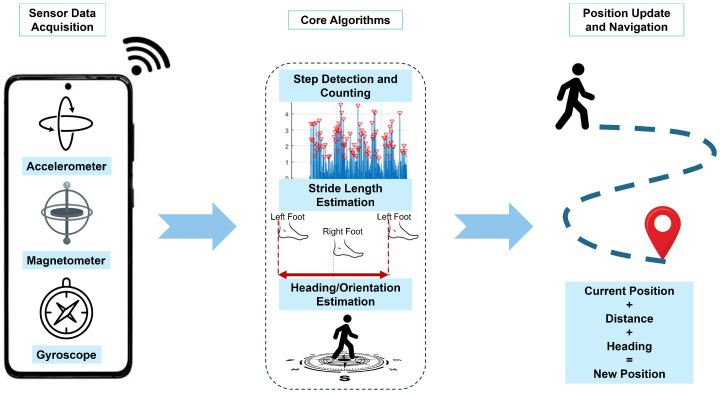
Pedestrian dead reckoning framework.

Pedestrian dead reckoning (PDR) is a popular solution among the different indoor localization techniques, since it is infrastructure-free and is compatible with off-the-shelf smartphones. PDR is an independent navigation technique that allows a smart device to track pedestrians’ movements by starting from a known location and reckoning their new positions based on their steps and directions. Unlike GNSS, which utilize satellites for location estimation, PDR estimates the location using only the smart device’s inertial sensors. As illustrated in Figure 1, the PDR framework transforms the raw inertial data into an estimated trajectory through four fundamental phases as follows [1]:

1. Inertial data acquisition from the smart device/smartphone. The accelerometer measures the linear acceleration of the smartphone, the gyroscope tracks the rotation, and the magnetometer is used to determine the local direction of magnetic north.
2. Step detection and counting. The system analyzes the inertial data and detects the steps taken by the pedestrian. Then, the distance walked is estimated using stride length estimation (SLE), which is usually based on the pedestrian’s gender and height.
3. Heading/orientation estimation. The system determines the direction that the pedestrian follows. This is one of the most challenging phases since minor errors in direction can lead the estimated path to diverge from the actual path over time.
4. Position update and navigation. Finally, PDR updates the pedestrian’s location from the prior location by fusing the estimated distance and the direction.

Extensive research emphasizes the feasibility of PDR for indoor navigation. For instance, ref. [5] introduced a widely adopted step length model using an accelerometer for pedestrian tracking. Foot-mounted inertial navigation with zero-velocity updates (ZUPT) is proposed in [6], while smartphone-based PDR systems are analyzed in [7].

Despite its efficiency, PDR’s practical implementation remains challenging for two main reasons. Firstly, low-cost inertial measurement unit (IMU) sensors are highly susceptible to accumulated drift and noise, which causes the estimated trajectory to diverge from the actual path over time. Secondly, the system’s accuracy is dependent on attitude, i.e., the specific way in which a pedestrian carries their smartphone, e.g., handheld, in-pocket, or on-call. The present article addresses these limitations by presenting a comprehensive, attitude-unconstrained PDR framework. Unlike existing methods that treat each sensor as a single vector, our approach introduces the intelligent breakdown of IMU data into individual degrees of freedom. Furthermore, to eliminate heading drift without manual calibration, we propose the heading estimation axis mapping (HEAT-MAP) algorithm. This algorithm dynamically remaps the smartphone’s local sensor axes to a consistent Earth coordinate system, ensuring stable tracking regardless of how the smartphone is held. To ensure long-term stability, this PDR framework is integrated with an adaptive Wi-Fi fingerprinting mechanism that provides absolute spatial anchors to periodically correct cumulative positioning errors.

### 1.1. Problem Formulation

The fundamental problem addressed in the present work is the unconstrained indoor localization of a pedestrian, using off-the-shelf smartphones. It is characterized by the following challenges:

1. Smartphone Placement Variability. Traditional PDR algorithms usually assume fixed smartphone orientations. However, sensors’ readings significantly vary across different body placements, leading to step counting and heading estimation errors.
2. Cumulative Gyroscope Drift. MEMS sensors, specifically gyroscopes, suffer from noise and bias. Errors resulting from the integration cause the estimated trajectory to diverge from the actual path.
3. Coordinate System Inconsistency. Changes in the smartphone’s orientation cause misalignment with the pedestrian’s direction or the Earth’s coordinate system. These misalignments result in unstable heading estimation, and manual calibration is required.

The proposed system is evaluated across diverse environments, including the infrastructure-less indoor premises of the Cyprus University of Technology, a gymnasium, and an outdoors asphalt concrete surface. For absolute spatial correction, provided by the adaptive weighted *k*-nearest neighbors (AW*K*NN) algorithm, the infrastructure density is characterized by a Wi-Fi access point density supporting three to eight nearest neighbors during the online phase. Furthermore, the scope of this study is focused on navigation on flat surfaces, while the presence of vertical transitions, such as stairs or elevators, is not included in the current analysis.

To solve the challenges of attitude-unconstrained indoor navigation, the proposed system is developed to meet specific performance requirements to ensure reliable trajectory tracking. Specifically, the system aims to maintain an average error of less than 1%, with a maximum error not exceeding 12% for high noise placements, such as ankle-mounted devices. In terms of orientation, the system is required to achieve an average heading error of less than ≈1% for in-pocket placement and ≈2% in reading-mode placement. Finally, for absolute spatial correction, the fusion of Wi-Fi and PDR aims to reduce the mean positioning errors by 56–66% and median errors by 48–65% across two carrying modes, e.g., pocket and reading modes.

The goal of this research is to develop a system that maintains high trajectory accuracy with unconstrained attitude and without requiring prior user calibration.

### 1.2. Contributions

The main contributions of this research are summarized as follows:

- A robust step counting algorithm is presented that utilizes the breakdown of raw IMU data across three degrees of freedom, identifying the most accurate sensor combinations for various body placements.
- The HEAT-MAP algorithm is developed to overcome heading drift by dynamically remapping sensor axes to the Earth’s frame and ensuring a consistent coordinate system without requiring manual calibration.
- An adaptive hybrid positioning system is described that employs adaptive weights to combine PDR and Wi-Fi fingerprinting, prioritizing PDR for short-term continuity and Wi-Fi for global alignment.

This paper is an extended version of our paper published in the International Conference on Indoor Positioning and Indoor Navigation (IPIN) 2025 [8]; preliminary findings from this research have also been previously reported as works in progress (WiP) in the IPIN 2024 and 2025 proceedings [9,10]. More specifically, while previous works introduced localized methods for step counting [9], Wi-Fi fingerprinting [10], and heading estimation [8] in isolation, this article presents a unified, comprehensive PDR framework featuring the first formal derivation and validation of the HEAT-MAP algorithm as a core navigator, the introduction of a dynamic 84-combination IMU breakdown, and a novel adaptive weighted fusion strategy for absolute spatial correction. Compared to the conference publication [8], it contributes over 50% new material, including a review of the PDR state-of-the-art literature and a performance evaluation of the proposed integrated PDR solution, along with a critical discussion of the proposed system’s strengths and limitations. This integrated approach provides substantial performance gains, specifically demonstrating a significant reduction in both step counting and heading errors across challenging smartphone placements.

The remainder of this paper is organized as follows: Section 2 reviews the related literature and state-of-the-art developments in PDR systems. Section 3 provides a detailed explanation of the proposed algorithms, including step detection and counting, heading and orientation estimation, and the position update and navigation frameworks. The experimental results and performance evaluation of the developed algorithms are presented in Section 4. Section 5 provides an in-depth discussion and evaluation of the proposed PDR system, its current limitations, and future research directions. Section 6 concludes the paper.

## 2. Related Work

PDR has emerged as a primary indoor navigation solution owing to its infrastructure-independent nature and its reliance on embedded sensors integrated within modern smart devices, i.e., smartphones. However, the implementation of PDR faces two primary challenges: the inherent drift of low-cost IMUs and accuracy variability due to different smartphone placements. In this section, we review the state-of-the-art developments in PDR systems, with a particular emphasis on how the proposed methods address these challenges in the different phases of PDR: (i) step detection and counting, (ii) step length estimation, (iii) heading estimation, and (iv) position update and navigation.

### 2.1. Step Detection and Counting

High-accuracy navigation requires robust IMU data calibration to mitigate the recursive integration of sensor errors, which otherwise leads to significant cumulative divergence [7]. A primary challenge is the magnetometer’s susceptibility to hard-iron and soft-iron distortions from nearby ferromagnetic materials or internal electronic components. To address this, three-dimensional (3D) ellipsoid fitting algorithms are often implemented to map distorted magnetic readings, which form an irregular shape due to nearby ferromagnetic materials, into a normalized sphere to find a reliable absolute heading reference, thus enhancing the heading estimation reliability [7]. To manage arbitrary smartphone orientations, existing research often utilizes the vector magnitude of 3D accelerometer data rather than focusing on an individual axis [11,12]. This traditional approach, termed the single measurement (SGL) method, processes the three-axis signals as a single integrated value by calculating the magnitude as follows:

(1) $$a=\sqrt{a_{x}^{2}+a_{y}^{2}+a_{z}^{2}},$$

where $a_{x}$, $a_{y}$ and $a_{z}$ are the accelerometer *x*-, *y*- and *z*-axes, respectively. While the SGL method is computationally efficient, it often loses the unique motion properties found in the particular axes, especially during complex movements or varying smartphone placements.

Digital low-pass or moving average filters are then applied to the signals to remove high-frequency jitter, effectively isolating the lower-frequency periodic signals associated with actual walking [11,13]. Furthermore, techniques such as the Savitzky–Golay filter are employed to smooth data while preserving the specific shape of acceleration peaks, critical for accurate step identification [14]. Alternatively, fast Fourier transform (FFT) analysis operates by transforming time-domain data into a frequency spectrum; a step is only confirmed if the amplitude in the typical walking frequency range significantly exceeds the noise floor. While calibration improves the estimation accuracy, it often restricts the plug-and-play functionality, as it frequently requires initialization procedures or predetermined pedestrian movements.

The effectiveness of PDR is influenced by inherent inertial biases and the physical placement of the smartphone. To address these challenges, numerous methodologies are utilized for identifying the physical patterns of the human gait, some of which are discussed in this section. In foot-mounted systems, the zero-velocity updated (ZUPT) algorithm utilizes the stationary phase of the gait cycle to reset velocity errors. It operates by providing a critical window for IMU drift mitigation, which resets the integration of errors at each step and prevents the cubic growth of positioning drift over time [15]. For handheld or body-mounted systems, adaptive thresholding is commonly used to identify local acceleration peaks [11,16,17], with thresholds dynamically adjusting to walking speeds, e.g., 0.3 for steady walking vs. 0.6 for pocket carrying. Similarly, these systems often utilize motion periodicity [18]. Furthermore, temporal constraints can also be employed, which prevent false counts caused by accidental movements.

These algorithms enforce a minimum time threshold, usually in seconds, between consecutive steps [2,11]. While peak detection is commonly used in PDR, the authors in [19] argued that it is sensitive to random smartphone shaking, and they utilized segmental correlation for step identification by monitoring the periodic fluctuations for a smartphone’s linear acceleration. This method is used to overcome the sensitivity of peak detection to random smartphone shaking. It operates by dividing linear acceleration data into temporary segments and estimating a correlation coefficient between the two halves of the segment. A step is confirmed only if this coefficient exceeds a specific threshold and the current segment correlates strongly with the previous one, ensuring that the detected motion is truly periodic walking rather than an accidental movement.

Moreover, for the smartphone’s placement, e.g., texting, calling, or in-pocket, support vector machine (SVM) classifiers or machine learning techniques are used [20]. In particular, in-hand modes prioritize the acceleration magnitude, whereas back-pocket placements rely on attitude features like pitch and roll due to high angular motion [20]. For thigh-mounted systems, detection utilizes thigh angles as the smartphone remains stationary relative to the limb [7].

### 2.2. Step Length Estimation (SLE)

Once a step has been identified, displacement is estimated using empirical methods like the Weinberg model [5,21], which is widely used as a nonlinear approach that correlates the step length with the vertical acceleration variance within a single stride. According to the model,

(2) $$\mathrm{Distance}=\sqrt[{4}]{A_{max}-A_{min}}\cdot n\cdot k,$$

where $A_{max}$ and $A_{min}$ are the maximum and minimum acceleration measured in the *z*-axis during a single stride, *n* is the number of steps, and *k* is a constant for unit conversion, e.g., to meters or feet. Alternatively, the three-step constraint step length estimation (TCSLE) model [16], which applies a weighted average of the previous three steps to maintain continuity, can also be utilized. On the other hand, biomechanical models can use the maximum and minimum thigh angles reached during a stride to estimate the distance via inverted pendulum logic [7].

Deep learning architectures are increasingly utilized for feature extraction from raw IMU data, in addition to heuristic models. A hybrid convolutional neural network (CNN)–long short-term memory (LSTM) system can extract spatial features from accelerometer data, while LSTM units capture temporal dependencies to delineate step boundaries [22]. Alternatively, deep learning models that estimate the velocity directly from IMU signals, often using ultra-wideband (UWB) measurements as ground truth, are used in [23]. In order to further reduce drift, rapid loop detection (RLD) via a camera that recognizes visited locations is employed in [24], and map matching with a particle filter that rejects trajectories that penetrate the wall is used in [7].

### 2.3. Heading/Orientation Estimation

Heading estimation is a critical component of PDR, as it defines the directional vector of movement and directly determines the expected trajectory’s accuracy. However, it is inherently susceptible to instability and cumulative drift. To mitigate these effects, sensor fusion strategies are implemented.

Complementary filters are frequently employed to fuse data from an accelerometer, gyroscope, and magnetometer [11]. While a gyroscope tracks relative angular changes, a magnetometer provides an absolute heading reference; their fusion enables the elimination of sensor bias [25]. Furthermore, tilt compensation algorithms estimate the orientation based on the smartphone’s spatial attitude to maintain directional integrity [26]. In structured environments, heuristic drift elimination (HDE) further limits heading divergency by aligning the estimated trajectory with the dominant headings, such as parallel or orthogonal corridor paths [18]. The extended Kalman filter (EKF) operates by minimizing the residual between the estimated coordinates and the recursive inertial position to ensure trajectory alignment [27]. For nonlinear, quaternion-based orientation, the unscented Kalman filter (UKF) is utilized to estimate biases without the computational complexity of calculating a Jacobian matrix. If high magnetic interference is detected, the UKF dynamically increases the measurement noise covariance (*R*) to prioritize gyroscope data over unreliable magnetometer readings [28].

The fusion of tri-axial accelerometer, gyroscope, and magnetometer IMU data can be used to ensure stability [29]. To mitigate the magnetometer’s susceptibility to magnetic interference, such as hard-iron and soft-iron effects, a least-squares ellipsoid fitting method is utilized for pre-calibration [30]. The gradient descent algorithm (GDA) filter, which optimizes the state by iteratively minimizing the error between the observed gravity or magnetic field vectors and the predicted orientation, can be used to ensure stability in the presence of noise [11]. Similarly, some systems employ threshold-based feature extraction to classify motion states, such as stationary versus walking and linear versus turning movements [16]. For stationary phases, a zero-angular rate update (ZARU) is implemented to eliminate gyroscope bias, whereas linear movement enables a straight-line constraint to prevent heading drift [16].

The challenge of variable smartphone placement, i.e., in-pocket or hand-swinging, is a significant focus in recent research [26]. For in-pocket placements, an improved rotational approach (IRA) utilizes the orthogonality between the thigh’s rotation axis and the direction of progression [12]. By identifying the leg flexion interval, the algorithm estimates an average rotation axis, filtering irregular gait movements and resolving the 180° directional ambiguity [12]. In contrast, for the swing mode, a single-point (SP) sampling method is used. This approach samples the heading at the specific point where the smartphone’s *y*-axis aligns most closely with the horizontal plane, thereby mitigating the yaw fluctuations inherent in hand-swinging motion [26].

### 2.4. Integration with Wireless Technologies

The integration of wireless technologies is a fundamental technique used to overcome the inherent limitations of inertial sensor-based indoor positioning by providing absolute reference points to correct the cumulative drift of inertial sensors. While PDR provides high-frequency autonomous tracking, its efficiency is restricted to short-term positioning due to cumulative sensor drift. As a result, integration with external wireless technologies is essential to establish absolute spatial anchors for initial positioning and periodic error correction.

Wireless integration can be either dedicated or existing infrastructure [3]. Dedicated infrastructure commonly includes specific radio frequency identification (RFID) tags or UWB beacons designed solely for localization, while existing network infrastructure includes pre-installed Wi-Fi or cellular networks to reduce deployment costs. Nevertheless, wireless technologies may coexist, such as Bluetooth low energy (BLE), UWB, and RFID, creating a heterogeneous environment that offers diverse options for varying accuracy and cost requirements [31]. The ability to operate across platforms is an essential requirement. To maintain consistency between the iPhone operating system (iOS) and Android environments, a multilayered approach combining Wi-Fi fingerprinting, PDR, and BLE iBeacons is often implemented [32]. This approach typically employs an AW*K*NN algorithm for Wi-Fi fingerprinting, while BLE iBeacons provide anchor points in regions with weak Wi-Fi coverage.

Wireless technologies act as global references for indoor systems, providing the coordinates required to maintain drifting sensor data aligned with the real world. The GNSS signals provide the ground truth for initial positioning and heading before a pedestrian enters an indoor environment, where satellite signals are lost [33]. BLE beacons are frequently deployed to provide periodic corrections. Similarly, if a wireless signal—like Wi-Fi or a brief global positioning system (GPS) fix—becomes available, the system uses it to reset the PDR coordinates, thereby eliminating the accumulated distance and heading errors [34]. Wireless RSS signals are integrated into decision trees alongside accelerometer, barometer, and magnetometer data to identify specific indoor landmarks, such as elevators or stairs [35].

Several algorithms are utilized to estimate locations and fuse data from various sources. The fingerprinting technique, for example, involves matching real-time RSS measurements against a pre-collected radio map database [35]. Furthermore, in systems using BLE beacons, data from PDR and BLE are often fused using a particle filter (PF), where particles represent potential pedestrian locations propagated according to PDR motion models. BLE signals with higher RSS are utilized to update particle weights, effectively tracking and correcting the estimated path and suppressing PDR drift [34]. An EKF can also be utilized to fuse the relative displacement data from PDR with the absolute positions derived from Wi-Fi or BLE, providing a continuous, drift-corrected trajectory that is independent of specific hardware constraints [34,35]. Towards this end, UWB technology can provide high-precision measurements for PDR prediction adjustments. PDR provides the dead-reckoning prediction for the next position, while UWB measurements provide the update phase within the Kalman filter. This fusion ensures the high sampling rate of PDR while utilizing UWB to eliminate trajectory deviation [36]. PDR is also used to filter out UWB outliers caused by NLoS conditions, such as signal blockage by structural obstacles or human presence, ensuring trajectory smoothness. The authors in [36] introduced the algorithm to manage the mapping between received signal strength indicator (RSSI) and time difference of arrival (TDoA), optimizing signal correction during fusion to enhance the matching accuracy. The various wireless technologies that can be combined with PDR, with their specific performance characteristics, are shown in Table 1.

Despite the advantages of integration, several articles focus on achieving independence from wireless infrastructure due to environmental sensitivity and deployment costs. The authors in [37,40,41,42] emphasize self-contained systems using internal smartphone sensors (MEMS) to maintain tracking in wireless-denied environments, where external signals are unreliable due to multipath interference and indoor obstacles.

## 3. Materials and Methods

The proposed PDR system utilizes an unconstrained framework to transform raw inertial sensor data into accurate real-time trajectories. Firstly, the step detection phase employs the intelligent breakdown of sensor data to optimize data fusion for accurate step counting. Secondly, the HEAT-MAP algorithm dynamically determines the smartphone’s orientation relative to gravity and transforms the sensor axes into Earth coordinates. This ensures consistent heading estimation for an attitude-unconstrained smartphone. Finally, an adaptive weighted fusion mechanism is incorporated that integrates absolute spatial anchors from Wi-Fi fingerprinting to periodically correct the cumulative errors inherent in PDR. The proposed system is designed for infrastructure-less indoor environments. During the online phase of the AWKNN algorithm, the environment consisted of a Wi-Fi density of 3 to 8 nearest neighbors. Despite the presence of structural obstacles, typical of indoor environments, which cause NLoS conditions, the system effectively integrates the signals. To eliminate the hard-iron and soft-iron distortions caused by ferromagnetic materials, calibration techniques were employed. The current experiment validation focused on flat floors, specifically within the Cyprus University of Technology facilities, as well as a gymnasium and outdoors asphalt concrete surfaces, and no elevators or stairs were included.

### 3.1. Step Detection and Counting

For step detection and counting, we propose the intelligent breakdown of the smartphone’s IMU data into individual degrees of freedom, as developed in [9]; this is used in the present work as a core component of a larger navigation system. Accordingly, the system gathers raw data at a frequency of 100 Hz from four primary smartphone internal sensors: a tri-axial accelerometer (A), a linear accelerometer (L), a gyroscope (G), and a magnetometer (M). Instead of processing each sensor’s data as a single measurement, the algorithm splits each into its three axes (*x*, *y* and *z*), providing a total of nine different data streams. By analyzing axis-specific data, rather than merely total magnitudes, the algorithm captures additional gait signatures that are body placement-specific. To identify the optimal data fusion combination, Algorithm 1 evaluates various combinations of these axis-specific streams. Specifically, with nine combinations, the system utilizes 84 possible permutations to determine the most effective fusion of raw IMU data for gait analysis. Specifically, the raw data streams from the four primary sensors (accelerometer, linear accelerometer, gyroscope, and magnetometer) are split into their three spatial components (*x, y, z*), creating nine distinct data streams for three combinations at a time, i.e., accelerometer–linear accelerometer–gyroscope (ALG), accelerometer–linear accelerometer–magnetometer (ALM), linear accelerometer–gyroscope–magnetometer (LGM) and magnetometer–accelerometer–gyroscope (MAG). From these data streams, every possible 3-axis combination (i.e., 93=84) is processed. The algorithm identifies the specific combination that results in the minimum average error relative to the ground truth step count recorded by a clicker counter and verified by a video recording. This allows the system to adaptively select the optimum combination, i.e., ALG, ALM, LGM or MAG, for the different body positions, i.e., hand, arm, waist, leg and ankle.
**Algorithm 1** Step Counting Algorithm1:Input three sensor data streams at a time (i.e., ALG, ALM, LGM, MAG)2:Split each sensor data stream into three components (*x*-, *y*-, *z*-axes), thus providing a total of nine different sensor component data streams and 84 total possible combinations3:**for** iteration=1,2,…,84 **do**4:  Estimate the magnitude5:  Deduct the magnitude mean6:  Set the threshold to 1 standard deviation7:  Estimate the total number of steps (peaks above the threshold)8:**end for**

In an attempt to reduce the computational overhead and ensure compatibility with off-the-shelf smartphones, the system utilizes raw measurements without traditional preprocessing filters. The algorithm involves estimating the magnitudes of the selected axes and performing bias removal, i.e., local gravity at rest, by subtracting the mean from the calculated magnitude. Step identification is performed through a threshold-based peak detection approach. The dynamic threshold is defined as one standard deviation of the acceleration measurements, and all peaks that exceed this threshold are categorized as valid pedestrian steps. One standard deviation is chosen as the peak detection threshold to serve as a self-adaptive filter for varying signal-to-noise ratios (SNRs) across different body positions. Unlike a fixed-magnitude threshold, which may fail when moving the smartphone from a high-impact position (e.g., ankle) to a low-impact position (e.g., waist). The one-standard-deviation threshold dynamically scales with the intensity of the pedestrian’s gait and the noise of the specific sensor combination. By setting the threshold to one standard deviation, the algorithm effectively captures the pedestrian’s movements, while rejecting noise and minor jitter, ensuring robust step-counting performance across multiple experimental trials and various tested speeds.

(3) $$\sigma \left(X\right)=\sqrt{\frac{\sum_{i=1}^{N}{\left(X_{i}-\hat{X}\right)}^{2}}{N-1}}$$

where *N* is the number of measurements in the dataset, $X_{i}$ represents each of the values, and $\hat{X}$ is the mean of $X_{i},i=1,\ldots ,N$. The utilization of one standard deviation makes the algorithm self-adaptive, thus allowing it to scale based on the signal’s energy, which varies based on the smartphone placement.

Section 2 analyzes various step-counting methodologies, such as zero crossing, auto-correlation and standard peak detection, which are inefficient in attitude-unconstrained scenarios where the device’s orientation is not fixed. In the proposed algorithm, permutation-based peak detection is chosen over complex and computationally intensive methods such as machine learning or the fast Fourier transform (FFT) for the following reasons. Firstly, FFT methods require long windows, which cause latency, whereas peak detection allows for real-time step detection and counting. Secondly, while machine learning models provide high accuracy, they are often too computationally intensive for smartphones’ hardware. The proposed method utilizes a dynamic threshold (1σ) and an intelligent axis breakdown technique. This approach maintains the raw gait across 84 different sensor permutations, maintaining an average error of between 0.89 and 11.72%, regardless of whether the phone is in a pocket, held in the hand or attached to the ankle.

### 3.2. Heading/Orientation Estimation

**Figure 2:**
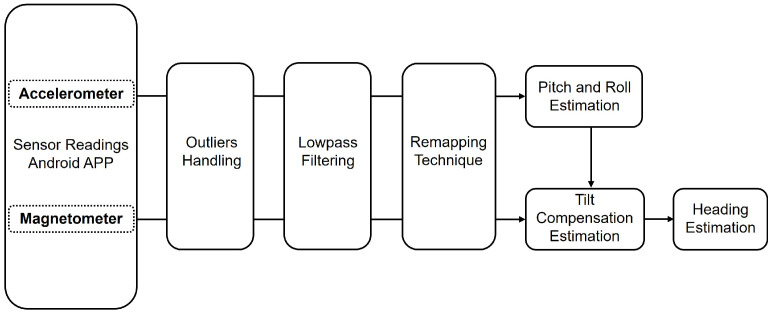
The HEAT-MAP algorithm. Reproduced with permission from [8].

The HEAT-MAP algorithm, developed in [8] and shown in Figure 2, is a low-complexity, real-time solution designed to mitigate sensor inconsistencies caused by variable smartphone placements; in the present work, it aids in providing linear, consistent heading estimation that serves the broader goal of eliminating drift navigation.

The HEAT-MAP algorithm utilizes the raw accelerometer (*a*) and magnetometer (*m*) sensor readings to estimate the heading (ψ) as follows:

(4) f(a,m)→ψ,

The preprocessing phase involves the identification and replacement of outliers, typically occurring during the initialization or termination of the application, with the respective axial mean values. Subsequently, a fourth-order Butterworth filter, utilizing a 100 Hz sampling frequency and a 0.1 Hz cutoff frequency, is applied to ensure a flat passband response and eliminate high-frequency noise. A sampling frequency of 100 Hz is established to ensure high-fidelity data acquisition that exceeds the Nyquist rate for all significant human movements. The selection of a 0.1 Hz cutoff frequency is specifically designed to isolate the quasi-static gravity vector from the dynamic accelerations associated with the human gait. While typical human walking occurs at frequencies between 1 Hz and 3 Hz, sudden movements and turns generate signals in the 0.1 Hz to 10 Hz range. By setting the cutoff at 0.1 Hz, the algorithm effectively eliminates the jitter caused by walking movements (1–3 Hz), while preserving the stable gravitational baseline for accurate pitch (θ) and roll (ϕ) estimation, calculated using the gravity acceleration of the *x*- and *y*-axes of the accelerometer, as shown in Equations (7) and (8), respectively. The core idea of the HEAT-MAP algorithm lies in its dynamic axis remapping. By calculating the standard deviation (σ), estimated using Equation (5) for the *x*, *y* and *z* accelerometer axes, the algorithm identifies the dominant axis corresponding to the gravity vector. Based on the axis dominance, estimated using Equation (6), the algorithm executes a remapping procedure to align the smartphone’s local frame (*x*, *y*, *z*) with the real-world orientation, i.e., side, forward and gravity (*x*’, *y*’, *z*’). This transformation follows the mapping *v’ = Mv*, where *M* is a selection and sign correction matrix derived from the magnetometer’s polarity. Following axis mapping, the pitch (θ) and roll (ϕ) angles are estimated and utilized to transform the magnetometer readings into the Earth-fixed coordinate system. These angles are utilized by the rotation matrices *R*(θ) and *R*(ϕ) to transform the magnetometer readings into the Earth-fixed frame (*m_x_, m_y_*). The final heading (ψ) is estimated using Equation (9). This methodology leads to a linear, consistent heading estimate that minimizes the drift typically associated with gyroscope-integrated systems.

(5) $$\sigma_{k}=\sqrt{\frac{\sum_{i=1}^{N}{\left(a_{k,i}-\hat{a_{k}}\right)}^{2}}{N}},k\in \left\{x,y,z\right\},$$

(6) $$D=\arg\max_{k}\left(\sigma_{k}\right),$$

(7) $$\theta =arctan\left(\frac{a_{x}}{\sqrt{a_{y}^{2}+a_{z}^{2}}}\right),$$

(8) $$\phi =arctan\left(\frac{a_{y}}{\sqrt{a_{x}^{2}+a_{z}^{2}}}\right),$$

(9) $$\psi =arctan\left(\frac{m_{y}}{m_{z}}\right).$$

On the other hand, the algorithm presented in [10] utilizes a frequency-domain analysis method designed to be attitude-unconstrained. This approach utilizes three fundamental phases: multi-axis rotation optimization, coordinate transformation with filtering and geographic projection. The optimization phase identifies the optimal coordinate system through a three-axis rotation, utilizing a grid search method to explore the global solution space. Specifically, *x*-axis rotation ($\theta_{x}$) between 0° and 90° is used to identify the peak step frequency; then, *y*-axis ($\theta_{y}$) and *z*-axis ($\theta_{z}$) rotations are performed to maximize the frequency ratio on the respective axes. During the coordinate transformation phase, the smartphone’s frame is realigned such that the primary axis corresponds with the pedestrian’s forward walking direction. The rotation is virtual and involves the numerical transformation of the smartphone’s local sensor frame into a new, optimized frame. The grid search explores a global solution, virtually rotating the sensor data across the *x*-, *y*- and *z*-axes ($\theta_{x}$, $\theta_{y}$, $\theta_{z}$) and estimating the alignment that results in the motion features. This process does not rely on a single sensor axis, and searching for the coordinate system alignment that maximizes the correlation between the local magnetic field and the forward walking direction ensures absolute directional integrity, even in the presence of magnetic disturbances. A low-pass filter is applied to the forward directional signal, while band-pass filters are implemented for lateral and vertical directions to isolate gait-related frequencies.

### 3.3. Position Update and Navigation

To prevent the inherent accuracy degradation of PDR, Wi-Fi fingerprinting is integrated as an absolute positioning correction mechanism [10]. The fingerprinting technique consists of an offline calibration phase and an online localization phase. During the offline phase, a radio frequency (RF) database of the indoor environment is constructed by collecting RSSI measurements along the trajectory at 2 m intervals, with a higher density of 1 m near endpoints. To account for signal fluctuations, stationary RSSI samples were acquired for 20–30 s at various intervals for each point. During the online phase, real-time position estimation is executed using an adaptive weighted *k*-nearest neighbors AW*k*NN algorithm. This method assigns weights to media access control (MAC) addresses based on their spatial detection frequency, using the weighted distance Equation (10), categorized as characteristic MACs (detected at <20% of locations; weight is set to 3.0), regional MACs (20–60%; weight is set to 1.5) and common MACs (>60%; weight is set to 1.0). The characteristic MACs are prioritized due to their high location specificity.

(10) $$d=\sqrt{\sum_{i}w_{i}\times {\left({\mathrm{RSS}}_{\mathrm{online},i}-{\mathrm{RSS}}_{\mathrm{fp},i}\right)}^{2}},$$

where $RSS_{online,i}$ represents the real-time signal strength of the *i*-th WiFi access point, $RSS_{fp,i}$ denotes the pre-stored signal strength of the same access point in the fingerprint database, and $w_{i}$ is the adaptive weight assigned to each access point based on its spatial coverage rate.

The system dynamically determines the optimal number of nearest neighbors (*k*), typically ranging from 3 to 8, based on the signal variance and matching quality. This is followed by a virtual rotation optimization that does not rely on a single sensor axis; instead, it mathematically searches for the coordinate system alignment that maximizes the correlation between the magnetic field and the forward walking direction. Long-term drift is corrected via adaptive weighted fusion, which utilizes absolute Wi-Fi spatial anchors to periodically reset the heading estimation. The final navigation solution is derived from the adaptive weighted fusion of PDR and Wi-Fi estimations as follows:

(11) $$P_{f}=\alpha \cdot P_{PDR}+\left(1-\alpha \right)\cdot P_{WiFi},$$

where $P_{f}$ represents the fused position estimation, $P_{PDR}$ represents the PDR position estimation, $P_{WiFi}$ represents the Wi-Fi position estimation, and α represents the adaptive fusion weighting coefficient.

The weighting coefficient α is formulated as a piecewise constant function of the Euclidean distance $d=\left|\right|P_{PDR}-P_{WiFi}{\left|\right|}_{2}$ between the two position estimates:

(12) $$\alpha \left(d\right)=\alpha_{\mathrm{base}}+\sum_{i=1}^{3}\Delta \alpha_{i}\cdot H\left(d-d_{i}\right)$$

where H(·) denotes the Heaviside step function. The parameters are specified as follows: $\alpha_{\mathrm{base}}=0.55$, $\left(\Delta \alpha_{1},\;d_{1}\right)=\left(0.10,\;5\;m\right)$, $\left(\Delta \alpha_{2},\;d_{2}\right)=\left(0.10,\;10\;m\right)$ and $\left(\Delta \alpha_{3},\;d_{3}\right)=\left(0.20,\;20\;m\right)$, yielding α∈{0.55,0.65,0.75,0.95} for the distance intervals d∈[0,5), [5,10), [10,20) and [20,∞), respectively.

Threshold selection is grounded in the error statistics of the Wi-Fi positioning module. Denoting the Wi-Fi positioning root mean square error (RMSE) as $\sigma_{\mathrm{WiFi}}$ (empirically measured as approximately 2.5–3.0 m in our experimental environment), the three thresholds correspond to approximately $2\sigma_{\mathrm{WiFi}}$, $4\sigma_{\mathrm{WiFi}}$ and $7\sigma_{\mathrm{WiFi}}$, respectively. When $d<d_{1}$, the discrepancy lies within the normal Wi-Fi noise range, and the system assigns nearly balanced weights to both sources. As *d* increases beyond successive thresholds, the system progressively shifts its reliance toward PDR to mitigate the impact of degraded Wi-Fi observations. For the coefficient increments, we adopt a progressive escalation strategy: moderate increments (Δα=0.10) at intermediate discrepancies preserve Wi-Fi’s global correction capabilities, while a larger increment (Δα=0.20) at extreme discrepancies enables rapid transition to a PDR-dominant mode to prevent trajectory discontinuities.

The fusion coefficient (α) dynamically shifts the system’s reliance between relative and absolute positioning based on the Euclidean distance between the two estimates. Specifically, if the variance is minimal (<5 m), the system prioritizes the absolute Wi-Fi reference (α = 0.55) to maintain global alignment. On the other hand, if the divergence is more than 20 m, the system trusts the PDR’s continuous relative displacement (α = 0.95) to prevent trajectory jumping while the Wi-Fi signal is stabilizing. Finally, the geographic projection phase maps the aligned body frame onto a geographic reference system. A quaternion-extended Kalman filter-based attitude and heading reference system (QEKF-AHRS) is deployed to fuse data from the accelerometer, gyroscope, and magnetometer. The QEKF dynamically prioritizes gyroscope data over unreliable magnetometer readings to maintain trajectory integrity. The resulting direction cosine matrix (DCM) derived from the estimated Euler angles describes the smartphone’s orientation relative to the Earth’s east, north, up (ENU) coordinate system.

## 4. Results

This section presents the evaluation and performance analysis of the proposed PDR system. To validate the robustness of the algorithm, the results were derived from different experimental settings involving various smartphone placements, such as handheld, in-pocket, and different walking speeds. In particular, for step detection and counting, the accuracy of the axis breakdown method is evaluated against that of the single measurement method, demonstrating its exceptional performance across different body positions and walking speeds. The experiments were carried out using off-the-shelf smartphones equipped with MEMS sensors. Data were gathered at a constant frequency of 100 Hz from the sensors. Moreover, the effectiveness of the HEAT-MAP algorithm is evaluated by examining the RMSE for heading estimates across ten different realistic smartphone placements, demonstrating its ability to mitigate directional drift without utilizing the gyroscope. Finally, the PDR’s overall positioning accuracy was validated through real experiments conducted in a 54 m *Z*-shaped corridor at the Cyprus University of Technology, demonstrating the PDR system’s stability and drift-correcting capabilities.

### 4.1. Step Detection and Counting

**Figure 3:**
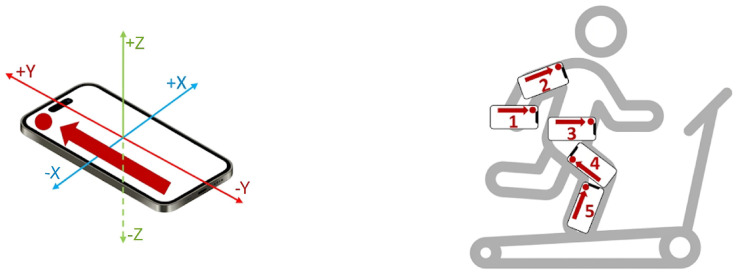
Smartphone placement. Reproduced with permission from [9].

For the evaluation of the proposed step detection and counting algorithm [9], the designed experiments used off-the-shelf smartphones placed at various body positions and a professional gym treadmill operated at different walking speeds, as shown in Figure 3. The system utilized five commercial smartphones, namely the Mi 11 Lite 5G, Mi 11i, and Poco X3 NFC (Xiaomi Technology Co., Ltd., Beijing, China); the P10 Lite (Huawei Technologies Co., Ltd., Shenzhen, China); and the 6X (Honor Device Co., Ltd., Shenzhen, China), placed on the hand, arm, waist, leg and ankle, respectively, and four smartphone sensors: a tri-axial accelerometer (A), a linear accelerometer (L), a gyroscope (G), and a magnetometer (M). Instead of using integrated vector magnitudes, the algorithm breaks down each of the four sensors into its three individual axes (*x*, *y* and *z*), creating nine distinct data streams for analysis. A fourth-order Butterworth filter with a 0.1 Hz cutoff frequency is applied to the sensor readings to eliminate high-frequency noise while maintaining a flat passband response. To ensure data quality and minimize external bias, the experiment was implemented in a controlled environment. In particular, three non-athlete participants, two women and one man, aged between 18 and 50, participated, equipped with five different smartphones simultaneously. The smartphones used were secured in water-resistant cases at five different locations, i.e., arm, hand, waist, leg and ankle. Each participant walked for 15 min at three different speeds, i.e., slow (3.3 km/h), normal (4.6 km/h) and fast (5.9 km/h), on a professional gym treadmill. The pedestrians’ steps were manually counted using a hand clicker and verified via video recordings from an iPad Pro (Apple Inc., Cupertino, CA, USA).

The results demonstrated that the proposed algorithm (OPT), which breaks the sensors’ readings down into individual axes, significantly outperformed the traditional single measurement method (SGL), as shown in Table 2. The SGL method treats the data from a sensor’s three individual axes (*x*, *y* and *z*) as a single combined measurement, rather than analyzing them separately. The best performance, with the lowest average error of 0.613%, was achieved by deploying the smartphone on the pedestrian’s arm at a slow speed using a combination of the accelerometer (*y*- and *z*-axes) and the linear accelerometer (*z*-axis). The arm was identified as the optimum body position for tracking.

Moreover, from the presented results, it becomes evident that both the physical location of the smartphone and the pedestrian’s speed can significantly impact the reliability of step detection. In particular, at normal speeds, the optimum combination (linear acceleration (*x*-axis), gyroscope (*z*-axis) and magnetometer (*z*-axis)) resulted in an average error of only 0.887%. The ankle was found to be the most challenging placement for accurate step counting, resulting in the highest overall average error across all tested speeds.

### 4.2. Heading/Orientation Estimation

**Figure 4:**
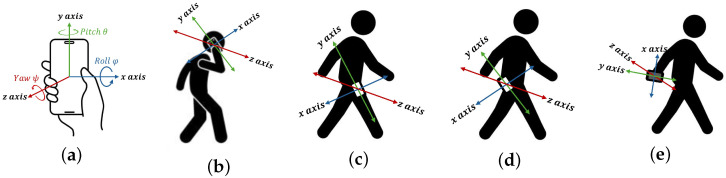
Smartphone positioning experimental settings: (**a**) in-hand, screen reading; (**b**) on-call, held to the ear; (**c**) in-pocket, front pants pocket; (**d**) in-pocket, rear pants pocket; and (**e**) swaying, right hand. Reproduced with permission from [8].

While step detection and counting (Section 2.1) is evaluated across broad body positions, this section expands the “hand” and “pocket” categories into ten specific orientations to evaluate the HEAT-MAP algorithm’s robustness against attitude-induced heading drift. Specifically, the accuracy and robustness of the HEAT-MAP algorithm [8] were evaluated in an experiment with ten different real-world smartphone placements, using the commercial smartphone model Poco X7 carried by a single pedestrian in all placements, walking on an asphalt concrete surface. Specifically, the ten realistic experimental settings were in-hand, screen reading, on-call, held to the ear (right and left side), in-pocket, right front pants pocket (screen facing body and outwards), in-pocket, left front pants pocket (screen facing body and outwards), in-pocket, right rear pants pocket (screen facing body and outwards) and swaying, right hand (screen facing body), as shown in Figure 4.

An Android smartphone was used to collect tri-axial accelerometer and magnetometer data at a 100 Hz sampling frequency. A commercial application, Sensor Logger [43,44], recorded the raw data in CSV format, which were then analyzed using MATLAB R2023a (version 9.14.0, Update 8). A pedestrian used a military magnetic compass to establish a static ground truth heading of 220° prior to the trials. During the walks, a dynamic tolerance of ±10° was maintained for the natural gait variations inherent in human locomotion. This interval ensured that the obtained RMSE values represented the system’s ability to track the intended path while considering the flexible body movements of a pedestrian. The HEAT-MAP algorithm was evaluated based on its root mean square error (RMSE) compared to the true heading derived from the military compass and the heading estimated from the commercial application. The results, shown in Table 3, proved that the algorithm successfully estimated the pedestrian heading, with an RMSE of less than 14.67° across all tested experimental settings.

It was also concluded that the accuracy of heading estimation is closely tied to the dominant axis, which indicates gravity, and the stability of the smartphone’s position. Specifically, the in-pocket, left front pants pocket (screen facing outwards) position was determined to be the most accurate position for navigation, resulting in the lowest RMSE of 1.75°. However, the on-call, held to the ear position yielded the highest errors, i.e., 13.78° to 14.67°. This may be due to muscular activity, tremors, head movements and facial movements during conversation. The axis performance is another significant finding; in particular, smartphone placements where the *x*-axis was dominant provided the lowest RMSEs, while the *z*-axis dominance resulted in the highest errors. In addition, the HEAT-MAP algorithm achieved relatively constant performance by relying only on the fusion of accelerometer and magnetometer data, rather than the gyroscope, which was prone to bias, thus avoiding cumulative drift issues, which typically degrade PDR’s accuracy over time.

### 4.3. Position Update and Navigation

Aiming to provide absolute positioning correction and eliminate the cumulative errors present in sensor-based tracking, in [10], PDR was fused with Wi-Fi technology. The experiment was conducted in a 54 m *Z*-shaped corridor at the Cyprus University of Technology. The experiment was performed by a single healthy adult male participant (177 cm, 70 kg), who maintained a continuous and steady gait throughout, without stopping or obstacle avoidance maneuvers. Normal pedestrian activity was present during both the offline fingerprint collection and online testing phases, reflecting realistic indoor deployment conditions.

**Figure 5:**
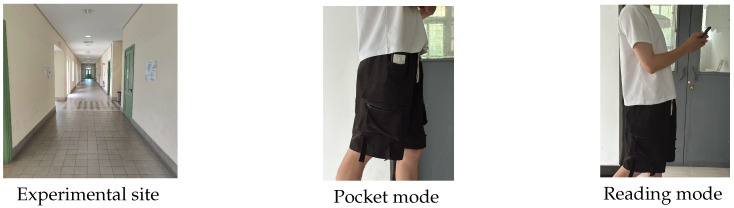
Experimental site, pocket and reading modes. Reproduced with permission from [10].

The Wi-Fi fingerprinting offline phase lasted approximately 10 h before the online testing. The fingerprints were collected at 2 m intervals along the path, with 1 m intervals near endpoints. To account for signal variations, multiple 20–30 s stationary measurements of the RSSI were taken at each point during different time periods. During the online phase, the pedestrian walked at normal speeds (1.2–1.5 m/s), using a commercial smartphone. The experiment included two carrying modes, pocket and reading modes, as shown in Figure 5. Three independent trials were conducted for each carrying mode at different time periods. These trials were primarily designed to evaluate the feasibility and consistency of the heading estimation module, which is the dominant factor affecting the overall PDR accuracy in attitude-unconstrained scenarios. Following an evaluation of the algorithm’s functional consistency, a single session in which both carrying modes were recorded under the same temporal conditions was selected for subsequent processing. However, we also recognize that evaluating the complete fusion pipeline independently across all trials would provide a more comprehensive assessment of system repeatability, and a more thorough systematic evaluation will be conducted in future work.

The real-time RSSI data were matched against the database using adaptive weights. The integration of Wi-Fi significantly improved the navigation accuracy and stability compared to using PDR or Wi-Fi alone. As shown in the experimental results—Table 4—the proposed method maintains high accuracy across varying carrying modes. The algorithm improves upon the classical Weinberg model [5] by incorporating a multi-factor adjustment mechanism, which considers the pedestrian’s height to set a “base” step length and then dynamically adjusts it based on acceleration variations and the step frequency. The actual numbers of steps taken in both evaluated carrying modes, i.e., reading and pocket modes, were correctly identified as 92 and 89 steps, respectively. The detected steps were consistently distributed throughout the time series, which confirmed the algorithm’s robust stride recognition capabilities, even with non-stationary walking signals. In addition, the adaptive threshold effectively managed different signal amplitudes caused by varying walking behaviors. Specifically, the threshold was approximately 0.3 and 0.6 for reading and pocket modes, respectively. Furthermore, pocket mode achieved the best performance, with an average error of 0.66 m and a 95th percentile error of 1.4 m, while, for reading mode, an average error of 1.10 m and a 95th percentile error of 2.82 m were achieved, as shown in Table 4.

**Figure 6:**
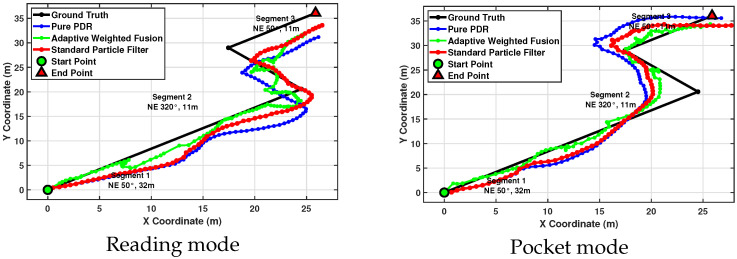
Positioning results. Reproduced with permission from [10].

The fusion of Wi-Fi and PDR reduced the mean positioning errors by 56–66% and the median errors by 48–65% across both carrying modes, as shown in Figure 6. While standalone Wi-Fi positioning provided poor stability, i.e., 95th percentile errors of up to 4.78 m, its fusion with PDR successfully eliminated the impact of attitude variations, i.e., switching from hand to pocket, on the overall accuracy. The adaptive weighted fusion outperformed the standard particle filter method, which had mean errors ranging from 1.36 m to 1.52 m.

Moreover, the algorithm achieved an average heading error of 16.91°, whereas pocket mode exhibited a higher average error of 22.74°. The overall heading change trends were accurately estimated throughout the 54 m test path, which included two significant 90° turns. It successfully identified the forward direction axis of the smartphone by searching for the maximum peak in step frequency in the frequency domain, allowing for orientation-independent tracking.

### 4.4. Comparative Performance Analysis

To evaluate the efficacy of the proposed system, the experimental results are compared with the results in [7]. The authors utilized a pocket-worn smartphone for PDR, focusing on a frequency-domain approach to estimate the step length and using multi-sensor fusion for heading. As shown in Table 5, the proposed method outperforms Zhao et al.’s system in terms of both detection accuracy and operational flexibility. While the authors in [7] achieved stride detection sensitivity of 99.18%, thus representing an error of 0.82%, specifically for a pocket-worn smartphone, our proposed optimum fusion (OPT) method achieves a lower step counting error of 0.613% for arm-mounted devices, while maintaining robustness across five distinct body placements. While the authors in [7] reported a slightly lower average position error in rectangular walking trajectories, their system is highly dependent on the pocket-worn constraint. In contrast, our PDR system maintains high-level performance without any attitude constraints, making it significantly more adaptable to real-world pedestrian behavior, where smartphones are rarely fixed. By utilizing the HEAT-MAP algorithm and adaptive Wi-Fi-PDR fusion, our system effectively mitigates the long-term cumulative drift that typically affects standalone PDR systems, such as the one proposed by the authors in [7], providing a more reliable solution for diverse indoor environments.

## 5. Discussion

The experimental results demonstrate that the proposed attitude-unconstrained PDR framework provides a significant improvement in both accuracy and robustness compared to traditional PDR across various operational conditions. The fusion of Wi-Fi and PDR overcomes the limitations of the standalone PDR systems described in [9,10].

The system’s main strength is its robustness to smartphone placement, which is achieved through the optimum fusion of raw IMU data and the HEAT-MAP algorithm. An important finding of this research is that breaking down tri-axial fusion data into individual degrees of freedom, rather than treating each sensor as a single magnitude (SGL), significantly improves the accuracy. While the SGL method exhibited errors as high as 35.7% for various leg placements, our proposed method resulted in errors of between 4.5% and 6.7%. The evaluation of 84 sensor combinations identified that the optimum configuration, which included the utilization of the accelerometer-*y*, accelerometer-*z* and linear accelerometer, achieved a minimum average error of 0.613% for arm placements.

The HEAT-MAP algorithm, on the other hand, effectively mitigates heading drift by dynamically remapping sensor axes to Earth coordinates. By identifying the dominant gravity axis through standard deviation analysis, the system maintains consistent orientation regardless of whether the smartphone mode is in-pocket, on-call or swaying in the hand. This approach successfully avoids the cumulative bias of traditional gyroscope-based PDR. In particular, in-pocket mode was identified as highly accurate for navigation, with an RMSE of 1.75°, while, even in the challenging on-call scenario (subject to facial tremors), our system outperformed existing benchmarks, with an RMSE of 14.67°. Furthermore, the HEAT-MAP algorithm demonstrated outstanding robustness by maintaining directional integrity without relying on the gyroscope, which was affected by temporal drift. By dynamically remapping the sensor axes to the Earth’s frame based on gravity vector dominance, the system effectively mitigated the heading errors that usually arise when a pedestrian changes placements, such as switching between screen reading, on-call or in-pocket modes.

Moreover, the reliability of the proposed system in indoor navigation is attributed to its adaptive weighted fusion mechanism, which mitigates the inherent drift of PDR by integrating absolute spatial references from Wi-Fi fingerprinting. Unlike other fusion models, this approach dynamically adjusts the system’s dependency on each technology based on the real-time environmental conditions and signal reliability. The core function is the fusion coefficient (α), which switches between relative and absolute positioning estimates based on their spatial variance and provides high-confidence alignment. When the Euclidean distance between the PDR estimate and the Wi-Fi estimate is minimal, namely less than 5 m, the system classifies the Wi-Fi signal as stable. In these instances, the coefficient is set to α = 0.55, increasing the weight of the absolute Wi-Fi reference to correct any minor cumulative drift in the PDR trajectory. Moreover, in scenarios where the Wi-Fi signal diverges significantly, mostly due to multipath effects or temporary signal loss, the system automatically shifts its trust toward the PDR’s relative displacement. By increasing the coefficient to α = 0.95, the system effectively ignores incorrect trajectory jumping—a common phenomenon in wireless systems—and maintains a smooth, continuous path based on inertial data while the Wi-Fi signal stabilizes. The experimental evaluations concluded that the system reduced the mean positioning error by 56–66% and the median error by 48–65% across various carrying modes compared to PDR alone. It demonstrated exceptional precision in common real-world scenarios. For instance, in pocket mode, it achieved an average error of only 0.66 m, while, in reading mode, it maintained sub-meter median accuracy at 0.84 m.

While the experimental results demonstrate the high precision and attitude-unconstrained behavior of the proposed PDR system, it is important to acknowledge certain limitations of this study. Firstly, the performance superiority of our system—specifically the comparison between the 84-combination IMU breakdown and the results reported by Zhao et al. [7] in Table 5—is primarily established through an inter-study analysis. While this serves as a benchmark for evaluating relative efficiency gains, it is important to highlight that these results were not obtained under identical experimental conditions due to differences in specific hardware platforms, sensor specifications and databases. Secondly, the HEAT-MAP heading estimation algorithm was initially validated in a controlled outdoor environment to establish a baseline under minimal magnetic interference. Since magnetometer measurements are susceptible to indoor magnetic interference from metal structures and electronic devices, specialized indoor verification of the heading estimation remains an area for future research. Thirdly, during the experiments, true heading values were established using a military magnetic compass, which resulted in the reported RMSE values being inherently tied to the accuracy and calibration of this reference baseline. Therefore, the performance metrics reflect the system’s alignment with this reference, rather than an absolute, error-free ground truth. Moreover, the current experimental study focusing on indoor settings involved only a limited number of participants and specific environments, i.e., the Cyprus University of Technology facilities and a gymnasium. Therefore, the dataset may not fully reflect the variability in gait patterns found across a broader demographic, e.g., including elderly individuals or people with mobility impairments, or more diverse, crowded and complex indoor environments.

The aforementioned limitations open up new and promising opportunities for future research. Firstly, ankle placement remains a challenge, exhibiting the highest average error of 23.68%. This may be due to the quick accelerations experienced in the limbs, which can exceed the dynamic thresholds of the peak detection algorithm. Secondly, although the PDR framework is an infrastructure-less system, the offline phase of Wi-Fi fingerprinting is time-consuming, requiring the construction of a radio frequency database at 1–2 m intervals. It should be noted that the thresholds and coefficient increments in the adaptive fusion rule—Equation (12)—are calibrated based on the Wi-Fi positioning accuracy observed in the current experimental environment (RMSE ≈ 2.5–3.0 m). In environments with substantially different Wi-Fi positioning characteristics (e.g., sparse access point deployment or severe multipath conditions), these parameters may require recalibration. A more principled approach to multi-source fusion, such as factor graph-based optimization, which jointly models the uncertainty of each positioning source through probabilistic inference, could potentially eliminate the need for manual threshold design. This represents a promising direction for future work. Additionally, we are in the process of integrating Wi-Fi fine timing measurement (FTM) to enhance the positioning accuracy. The utilization of the round-trip time (RTT) to estimate distances to access points (APs) could provide meter-level precision, effectively overcoming the inherent instability of traditional RSSI-based methods.

In addition, heading estimation relies on a magnetometer, which is susceptible to hard-iron and soft-iron distortions in environments with ferromagnetic materials. Ongoing research is focused on improving the HEAT-MAP algorithm for complex indoor environments. This involves extensive inertial data collection in highly crowded areas with ferromagnetic structures to improve the algorithm’s resilience against magnetic interference. In particular, as part of our ongoing research, we are actively validating the performance of HEAT-MAP indoors, combined with an enhanced ZARU-based adaptive complementary filter specifically designed for managing magnetic environments. Preliminary results from this enhanced system, validated in a high-density shopping mall in Cyprus, demonstrate significant improvements in robustness; for instance, the integrated approach achieved an RMSE as low as 2.88° in use scenarios and effectively mitigated local magnetic interference through dynamic gain controllers. These findings provide a strong indication of the algorithm’s performance in real-world indoor conditions and will be reported in future research.

## 6. Conclusions

The presented attitude-unconstrained PDR system is designed to overcome the accuracy limitations inherent in low-cost IMU sensors and across varying smartphone placements. Instead of using conventional single-measurement approaches, the step detection algorithm utilizes the intelligent breakdown of raw IMU data into three degrees of freedom and identifies optimal sensor combinations that significantly enhance the system’s reliability. This approach achieved significant accuracy across multiple device placements and walking speeds, yielding a low average step counting error of 0.613%. Furthermore, the HEAT-MAP algorithm was developed to addressing the critical issue of orientation drift. By dynamically remapping the sensor axes to the Earth’s frame and fusing accelerometer and magnetometer data, rather than relying on the error-susceptible gyroscope, the algorithm maintained an RMSE of less than 14.67° across ten realistic experimental settings based on different phone placements. Moreover, by integrating an adaptive Wi-Fi fingerprinting technique, the system successfully mitigated the cumulative errors inherited by traditional PDR. This hybrid approach demonstrates significant efficiency through its stability in diverse modes, achieving mean positioning errors of only 0.66 m in pocket mode and 1.10 m in reading mode.

These findings effectively reduce the gap between the desired attitude-unconstrained smartphone use and the achieved PDR accuracy. However, the experimental sample was relatively small. To address the current limitations, ongoing and future research is focused on expanding the diversity of the testing groups and the variety of indoor environments, e.g., to include multi-floor buildings with elevators and stairs, as well as improving the HEAT-MAP algorithm for complex indoor environments. This involves extensive inertial data collection in highly crowded indoor places that contain ferromagnetic structures to improve the algorithm’s robustness against magnetic interference. The utilization of larger datasets will confirm the system’s reliability for real-world pedestrian behaviors. Moreover, the system is being evolved to integrate Wi-Fi FTM to enhance the positioning accuracy. The utilization of RTT for estimating distances to APs could provide meter-level precision, effectively overcoming the inherent instability of traditional RSSI-based methods.

## Figures and Tables

**Table 1:** Wireless technologies characteristics.

Technology	Measurement Methods	Accuracy	Advantages
Wi-Fi [3,31,35]	RSSI, Fingerprinting, RTT	3–10 m	Wide coverage, uses existing infrastructure
BLE [31,34,36]	RSSI, Fingerprinting, AoA	1–5 m	Low power, easy to deploy, periodic resets
UWB [3,31,33]	ToA, TDoA, AoA	Centimeter	High accuracy, insensitive to multipath, anti-jamming
Acoustic [31]	ToF, TDoA, DoA	Density-dependent	Good compatibility with standard hardware
RFID [3,33]	Proximity, RSS Analysis	Variable	Uses active/passive tags
GPS/GNSS [37,38,39]	Satellite Signaling	High (Outdoor)	Standard for outdoor initialization
Cellular [3,40]	Cell-ID, E-OTD	50–200 m	Wide availability via existing GSM/CDMA networks

**Table 2:** OPT vs. SGL.

Placement	OPT Average Error (%)	SGL Average Error (%)
Hand	0.887–7.423	9.166–15.727
Arm	0.613–2.258	10.447–15.226
Waist	1.861–3.722	7.520–17.168
Leg	4.532–6.758	24.685–35.784
Ankle	6.055–11.720	10.001–16.388

**Table 3:** Optimum axis mapping for heading estimation.

Smartphone Placement (see Figure 4)	Dominant Axis	HEAT-MAP RMSE (°)	Sensor Logger [43] RMSE (°)
In-hand, screen reading	*y*	9.91	36.18
On-call, held to the ear (right side)	*x*	13.78	51.88
On-call, held to the ear (left side)	*z*	14.67	62.62
In-pocket, right front pants pocket (screen facing body)	*z*	4.37	71.99
In-pocket, right front pants pocket (screen facing outwards)	*x*	11.14	25.96
In-pocket, left front pants pocket (screen facing body)	*y*	5.18	16.09
In-pocket, left front pants pocket (screen facing outwards)	*x*	**1.75**	40.41
In-pocket, right rear pants pocket (screen facing body)	*z*	6.22	20.33
In-pocket, right rear pants pocket (screen facing outwards)	*x*	7.22	72.06
Swaying, right hand (screen facing body)	*x*	10.42	63.32

**Table 4:** Positioning accuracy and errors.

Error	Pocket Mode	Reading Mode
Mean Error	0.66 m	1.10 m
Median Error (50%)	0.61 m	0.84 m
95th Percentile Error	1.41 m	2.82 m

**Table 5:** Comparative performance analysis.

	Zhao et al. [7]	Proposed Method
Smartphone Placement	Pocket-worn	Attitude-unconstrained (hand, arm, waist, leg, ankle)
Step Counting Error	0.82% (pocket-worn)	0.61% (arm)
Heading Estimation	Gradient Descent Algorithm (GDA)	HEAT-MAP (gyroscope-independent)
System	Standalone PDR	Adaptive Wi-Fi and PDR fusion
Mean Position Error (Rectangular Walking Trajectory)	1.00%	1.13%
PDR Scenarios	Pocket-worn smartphone only	Multiple scenarios

## Data Availability

The original contributions presented in this study are included in the article. Further inquiries can be directed to the corresponding author.
